# Mortality in Schizophrenia-Spectrum Disorders: Recent Advances in Understanding and Management

**DOI:** 10.3390/healthcare10122366

**Published:** 2022-11-25

**Authors:** Vaios Peritogiannis, Angeliki Ninou, Maria Samakouri

**Affiliations:** 1 Mobile Mental Health Unit of the Prefectures of Ioannina and Thesprotia, Society for the Promotion of Mental Health in Epirus, 45445 Ioannina, Greece; 2 Department of Psychiatry, Medical School, Democritus University of Thrace, 68100 Alexandroupolis, Greece; 3 “Skitali” Day Center, Society for the Promotion of Mental Health in Epirus, 45445 Ioannina, Greece

**Keywords:** cardiovascular disease, mortality, physical morbidity, schizophrenia-spectrum disorders, severe mental illness, suicide

## Abstract

Schizophrenia is a life-shortening disease and life expectancy in patients may be 15–20 years shorter than in the general population, with increasing longevity gap over time. Premature mortality in schizophrenia-spectrum disorders is mainly due to preventable natural causes, such as cardio-vascular disease, infections, respiratory tract diseases and cancer, alongside suicide, homicide and accidents. There is a complex interplay of factors that act synergistically and cause physical morbidity to patients and subsequent mortality. Smoking, alcohol/substance abuse and sedentary life style, alongside disease-related factors, such as metabolic abnormalities and accelerating aging contribute to physical morbidity. Moreover, the symptomatology of psychosis and stigma may limit patients’ access to quality medical care. Interventions to promote physical health in those patients should be multifaceted, and should target all patient-related modifiable factors, but also should address service-related healthcare disparities. Long-term antipsychotic use (including clozapine and long-acting injectables) is associated with substantially decreased all-cause mortality, including suicide and cardiovascular mortality, in patients with schizophrenia despite the well-known cardiometabolic adverse effects of second-generation agents. Integrated care may involve co-location of physical and mental health services, liaison services, shared protocols and information sharing systems, and has emerged as a way to address the physical health needs of those patients. Interventions to address mortality in schizophrenia and related syndromes should take place as early as possible in the course of the patients’ treatment, and could be an integral component of care delivered by specialized early intervention services.

## 1. Introduction

Mortality is considered as the most important outcome measure in medicine and a standard indicator of the performance of the health system and this is also the case in the evaluation of mental health policies and services [1]. Yet, according to previous evidence, life expectancy in patients with chronic and severe mental illness (SMI) is significantly shorter than in the general population. In a meta-review analysis of 20 systematic reviews, it was found that mental disorders pose an increased risk in all-cause mortality to patients compared with the general population, and patients’ life expectancy is reduced by 10–20 years [2]. Another meta-analysis of 148 studies estimated that 14.3% of deaths worldwide each year are attributable to mental disorders [3].

Among psychiatric disorders schizophrenia and related syndromes are still the most devastating, according to the recent Global Burden of Disease Study [4]. Schizophrenia is a severe and chronic mental illness, with generally modest prognosis and often poor long-term outcome [5,6]. The syndrome comprises a wide range of symptoms, such as positive and negative symptoms and cognitive impairment, that adversely affect social and occupational functioning of patients [7]. Antipsychotic drugs are the mainstay in treatment of schizophrenia and are particularly effective for positive symptomatology, but much less effective for negative and cognitive symptoms. Common adverse effects are extrapyramidal symptoms and tardive dyskinesia with first generation antipsychotics, whereas second generation agents are associated with higher rates of cardiometabolic side effects [8]. However, treatment of schizophrenia may be frequently undermined by patients’ non-adherence to medication and disengagement from mental health services [9,10,11]. 

Schizophrenia has been long considered to be a life-shortening disease [12], and recent evidence indicates that life expectancy in patients may be 15–20 years shorter than in the general population. Moreover, it is alarming that the gap is increasing over time, despite the substantial progress in the treatment of these disorders [13,14]. With regard to the causes of premature death in patients with SMI, previous research tended to overestimate the rate of unnatural deaths, that is suicide, homicide, and accidents [15]. Indeed, a large retrospective study suggested that most deaths that are over three-quarters, in patients with SMI were attributable to physical illnesses [16]. It is well documented that patients with psychotic disorders are at significantly higher risk for cardiovascular morbidity and mortality, compared to the general population. Moreover, in those patients, cardiovascular events are the commonest cause of death [17]. 

Addressing physical morbidity and mortality in schizophrenia-spectrum disorders should be an integral part of the holistic treatment of these disabling syndromes, and research on these topics is ongoing. The study of mortality in schizophrenia is also relevant for primary care providers and healthcare professionals. The aim of the present article is therefore to provide an up-to-date overview of the current state of knowledge regarding mortality in schizophrenia and related syndromes and to synthesize recent evidence from large nationwide studies and meta-analyses; to highlight the complex interplay among various risk factors; and to propose a practical framework for improving the management of medical comorbidity in those patients, that would be useful for everyday clinical practice across primary care and community mental healthcare settings. 

## 2. Mortality Rates in Schizophrenia-Spectrum Disorders

In a previous meta-analysis, it was shown that schizophrenia was associated with a weighted average of 14.5 years of potential life loss, that was higher for men than women (15.9 vs. 13.6, respectively). The overall weighted average life expectancy was 64.7 years and was lower for men than women (59.9 years vs. 67.6 years, respectively) [18]. In a nationwide study in South Korea, which processed data from 107,190 cases of patients with mental disorders (9387 patients with schizophrenia), over a 12-year period, it was found that patients with schizophrenia had a 2.4 times higher mortality risk compared to the general population [19]. Elevated mortality rates have been reported in first-episode schizophrenia patients as well. The 10-year follow-up of the ӔSOP first-episode cohort found that all-cause standardized mortality ratio was 3.6 times higher, compared with the general population [20], whereas a very recent meta-regression analysis of 76 studies found relative mortality to be higher in younger age groups [21].

## 3. Causes of Mortality

Despite major concerns regarding suicide in patients with schizophrenia, the so-called natural causes, mostly cardiovascular disease are accounted for the majority of deaths [22]. The most common causes of mortality in schizophrenia-spectrum disorders, both natural and unnatural, that are violent deaths, will be discussed below.

### 3.1. Unnatural Causes of Death

Unnatural deaths are accountable for the 20–25% of the total number of deaths in patients with schizophrenia in high-income countries, but also in those with low or middle income [15]. This category comprises suicide, homicide and accidents.

#### 3.1.1. Accidents

Accidental deaths in patients with schizophrenia seem to be more frequent than those due to suicide. In a large study in Sweden, it was found that the risk of death by accident was 2–4 times higher in patients than in the general population, a rate that was not fully explained by substance abuse comorbidity [23]. Similarly, in a more recent large nation-wide cohort study in Denmark, a significantly increased risk of accidental death among patients with schizophrenia, compared to the general population was found. The association of schizophrenia with accidental deaths appeared to be partly mediated by substance abuse, yet it was still 3-fold higher after adjusting for substance abuse [24]. Interestingly, a 15-year national retrospective cohort study in Brazil that included all patients hospitalized through the public health system for SMI, found that injuries, such as road accidents, had the highest excess mortality rate. This finding suggested that the pattern of mortality risk from specific conditions may differ between high- and low/middle income countries, probably due to social and economic adversities [25]. 

#### 3.1.2. Suicide

Suicide is the worst outcome in schizophrenia, with a lifetime rate in patients of approximately 5–10% [26,27]. Factors that have been associated with suicide risk in schizophrenia are affective symptoms, previous history of suicide attempts and the number of psychiatric admissions. Suicide risk in schizophrenia has been also related to younger age, closeness to illness onset, older age at illness onset, male gender, substance abuse and the period during or following psychiatric discharge [28]. Other demographic and psychosocial factors that increase the risk of suicide in individuals with schizophrenia include being unmarried, living alone, being unemployed, being intelligent, being well-educated, good premorbid adjustment or functioning, having high personal expectations and hopes, having an understanding that life’s expectations and hopes are not likely to be met, having had recent (i.e., within past 3 months) stressful life events, having poor work functioning, and having access to lethal means, such as firearms [27]. With regard to the impact of insight on suicide in schizophrenia, it has been suggested that both patients with poor and good insight in schizophrenia may attempt suicide. That is, patients with poor insight may do so under the influence of psychotic symptoms and probable command hallucinations, while patients with good insight may attempt suicide after the realization that they have suffered from schizophrenia and the stress, shame and stigma associated with the diagnosis [29].

#### 3.1.3. Homicide

Patients with SMI are more likely than the general population to be the victims of violent acts and there is a correlation of victimization with patients’ violent behavior, although it is not clear which precedes. Risk factors for victimization comprise young age, substance use, and psychotic symptoms, whereas the homeless patients are more likely to be the victims of violent acts [30]. In the aforementioned large Swedish study, patients with schizophrenia were found to have almost twice the risk of being victims of homicide, compared to the general population [31]. More recent evidence from Sweden yielded somewhat different results, as it was found that the diagnosis of schizophrenia, after adjusting for comorbid substance use and personality disorders had not been associated with the risk of being victim of violent acts [32]. In the above-mentioned nationwide study in Brazil patients with SMI, especially females, were found to be highly exposed to interpersonal violence and had an overall mortality relative risk from homicide 2.4 times higher than other patients [25].

### 3.2. Natural Causes

Most deaths in patients with mental disorders are attributable to natural causes. A previous meta-analysis found that a total of 67.3% of deaths among people with mental disorders were due to natural causes, 17.5% to unnatural causes, and the remainder to other or unknown causes [3]. This is also the case of patients with schizophrenia, in whom natural causes of death, mostly due to premature onset of serious medical diseases, are responsible for the increased mortality, and account for up to 80% of deaths [14]. Natural causes were mostly accountable for sudden unexpected deaths as well. In a recent autopsy study of cases of individuals with schizophrenia it was found that the majority of deaths (64.2%) were caused by natural diseases, whereas 12.0%, 11.5% and 9.7% of deaths were due to accidents, suicides, and homicides, respectively. In the remaining 9.7% of cases, the manner of death could not be determined [33].

#### 3.2.1. Cardiovascular Disease

The link between mental disorders and coronary heart disease has been highlighted in several studies, and it has been suggested to be bidirectional, that is both may cause one another [34]. Moreover, cardiovascular disease is the main cause of death in patients with psychotic disorders [35]. There is evidence that patients with schizophrenia die about 10 years earlier than the general population due to cardiovascular disease, including coronary heart disease [36]. According to a large-scale meta-analysis, which processed data from 3,211,768 patients and 113,383,368 controls, patients with SMI have 53% higher risk to suffer a cardiovascular disease; 78% higher risk to develop cardiovascular disease; and 85% higher risk of dying by cardiovascular disease compared to country-standardized general population [37]. Importantly, mortality due to cardiovascular disease in patients with schizophrenia did not decrease over the last decades, in spite of the general trend of decreasing mortality rates from cardiovascular disease worldwide [36].

**Figure 1:**
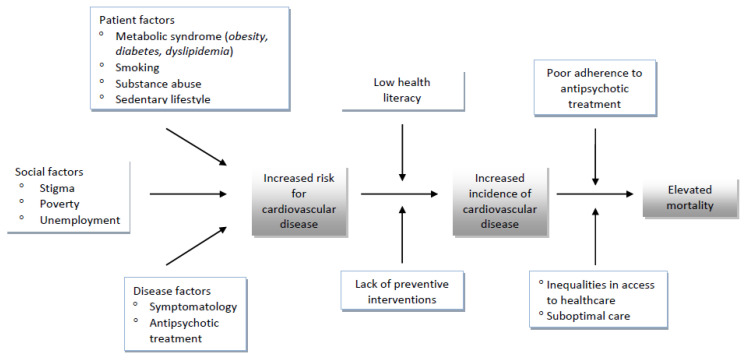
Contributing factors to the risk of mortality due to cardiovascular disease in patients with schizophrenia.

A recent systematic review of the literature on the outcomes of acute coronary syndrome in patients with schizophrenia yielded similar results. Mortality and major adverse cardiac events as well as stroke were more prevalent in patients with a schizophrenia diagnosis compared to those without. The difference could be partly explained by patients’ co-morbidities (e.g., diabetes, obesity) and patients’ habits, such as smoking and substance abuse. Additionally, schizophrenia patients received suboptimal care, in terms of revascularization, secondary prevention measures, including the prescription of statins, beta-blockers, and anti-platelets and follow-up, compared to patients without a psychiatric diagnosis [38]. Other factors such as poor health literacy, treatment nonadherence, substance abuse and lower socioeconomic status may also contribute to worse cardiovascular outcomes, whereas those patients may have several barriers to access the healthcare system and may experience disparities that undermine optimal care [39]. The interplay of factors that contribute to mortality from cardiovascular disease is depicted in Figure 1. 

#### 3.2.2. Respiratory Tract Diseases

Respiratory diseases are still more prevalent in people with SMI, compared with the general population. Studies consistently show a higher incidence of tuberculosis, chronic obstructive pulmonary disease, and pneumonia among patients with schizophrenia [17,40].

#### 3.2.3. Infections

In a recent meta-analysis, it was found that patients with schizophrenia had approximately 3-fold increased odds of hepatitis B and C, compared to the general population. The authors suggested that this association could be explained by behavioral factors, such as substance use and hazardous sexual behaviors. Or/and patients with schizophrenia may have an increased susceptibility to infections, due to abnormal immune cell function [41]. Infection-related mortality in patients with schizophrenia may be more relevant in low- and middle-income countries, as suggested by the nationwide Brazilian study, which found infections, such as tuberculosis, acute hepatitis, and HIV, to be associated with excess mortality risk in those patients [25]. 

Over the last two years there is a growing interest on the effects of the COVID-19 pandemic on patients with psychotic disorders. There is ample evidence that patients with schizophrenia have a high risk of being infected by the new coronavirus and display significantly higher mortality rates than the general population [42,43]. Accordingly, it has been suggested that those patients should be prioritized in vaccination programs [44], and there is some evidence that patients with mental illness may be as willing as the general population to be vaccinated against COVID-19 [45,46]. 

#### 3.2.4. Cancer

In a previous population-based study it was shown that compared with the general population, individuals with mental illness experienced excess mortality from most cancers. According to the authors, this could be accounted for by a higher prevalence of smoking, substance abuse, and chronic hepatitis B or C infections in individuals with mental illness. Moreover, excess mortality could also reflect late-stage diagnosis and/or inadequate treatment [47]. The study of the risk of cancer in patients with schizophrenia has yielded mixed results. In a recent meta-analysis, it was found that patients with schizophrenia have a higher risk of mortality from common site-specific cancers, particularly from breast, lung, and colon cancer [48]. Those findings differed from the findings of two other recent meta-analyses [49,50], whereas the most recent relevant meta-analysis reported a higher overall cancer risk for patients with psychotic disorders, relative to the general population [51]. It has been suggested that the mortality of different cancers may be influenced not only by cancer incidence but also by increased risk of suicide, unhealthy lifestyle, late-stage diagnosis, poor survival after diagnosis, and inadequate cancer treatment (e.g., surgery, chemotherapy, radiotherapy, endocrine therapy, and palliative care) [48]. A very recent UK-wide matched cohort study that used primary care records for the identification of cases, found that patients with schizophrenia had lower rates of cancer diagnosis but higher all-cause and cancer specific mortality rates following diagnosis, compared to those without a psychotic disorder. The authors suggested that the findings reflected barriers to cancer diagnosis and treatment in those patients [52]. Indeed, disruptions in cancer care may be common for patients with schizophrenia and are associated with adverse outcomes, such as cancer recurrence [53].

**Figure 2:**
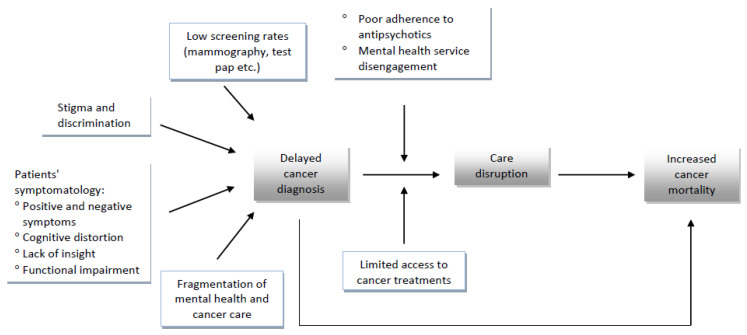
The interplay of factors that are associated with increased mortality by cancer in patients with schizophrenia.

Specific mention should be made for breast cancer in women suffering from schizophrenia. According to the most recent meta-analysis, antipsychotic use is moderately associated with breast cancer, and this could be partly mediated by prolactin-elevating properties of certain agents [54]. Other recent research has suggested that the risk is greater for cumulative exposure to prolactin-increasing antipsychotics in women with 5 or more years of exposure than those with less than 1 year. Importantly, no increased risk for exposure to prolactin-sparing antipsychotics, such as clozapine, quetiapine, and aripiprazole was found [55]. However, it has been suggested that there may be more than one physiological mechanism that could underlie the association between antipsychotic use and breast cancer, such as antipsychotic-induced obesity and metabolic syndrome, hyperlipidemia and diabetes mellitus [54]. The barriers in diagnosis and proper management of cancer in patients with schizophrenia are summarized in Figure 2. 

## 4. Contributing Factors to Increased Mortality

### 4.1. Smoking

The association of schizophrenia and smoking has been long recognized. Despite the global efforts for the reduction of smoking through campaigns and bans, patients with schizophrenia remain heavy smokers, with minimal changes over the decades [56]. It was thought for long that smoking was a secondary effect of the disorder itself, either as self-treatment effort, or as part of the institutionalization process of the patients. However, those hypotheses could not fully explain the relationship of smoking and psychotic disorders. More recently a reciprocal relationship has been proposed, according to which smoking is causally linked to the risk of schizophrenia, probably through a shared genetic vulnerability [57]. This is in line with the findings of a recent literature review, in which the reported range of prevalence of nicotine use during the prodromal phase of schizophrenia across studies was 16.6–46%. Interestingly, several studies reported an increased risk for psychosis in heavy smokers [58]. Notably, though recent evidence does not support the hypothesis that smoking improves cognitive symptoms in patients with schizophrenia, there is some evidence that it may improve negative symptoms and the extrapyramidal side effects of antipsychotics. It is worth mentioning that smoking can reduce the serum levels of certain antipsychotics, such as clozapine and olanzapine [56].

### 4.2. Alcohol/Substance Abuse

Patients with schizophrenia are particularly vulnerable to alcohol/substance abuse disorders, with well-known negative effects on outcome and health [59]. In a national retrospective longitudinal cohort study of patients with schizophrenia in the USA, comprising 1,138,853 patients, the misuse of alcohol or other drugs was one of the leading causes of death [40].

### 4.3. Obesity and Metabolic Syndrome

There is ample evidence that chronic patients with schizophrenia have high rates of obesity and metabolic syndrome, which is associated with increased cardiovascular morbidity and mortality. A previous meta-analysis found that the overall rate of metabolic syndrome in patients with schizophrenia was 32.5%, with only minor differences across countries and treatment settings, and no differences between genders [60]. Similar findings were recently reported on Chinese patients with schizophrenia [61], as well as on patients with schizophrenia in developing countries [62], although data are limited at present. Contributing factors to obesity are the side effects of antipsychotic medication, such as weight gain, metabolic abnormalities and sedation. Other associated factors are the sedentary lifestyle of patients and negative symptoms. Interestingly, a recent study in China on first-episode, drug-naïve patients with schizophrenia reported that the obesity rate of patients was similar to that of controls [63].

### 4.4. Diabetes Mellitus 

Schizophrenia is associated with increased risk for type 2 diabetes mellitus, resulting in elevated cardiovascular risk and increased mortality. The exact prevalence of type 2 diabetes among people with schizophrenia varies across studies [64] and ranges between 2- and 5-fold higher than in the general population, whereas the etiology is complex and multifactorial [65]. Several common diabetogenic factors are similarly encountered in the general population, such as obesity, poor diet and limited physical activity. Other disease-related factors are sedentary lifestyle, socioeconomic adversities, treatment side effects, and limited access to medical care, which contribute to the development of diabetes and undermine its management [65,66]. Schizophrenia itself is further considered as causal factor for diabetes, as implied by the higher prevalence of diabetes in newly diagnosed young patients with schizophrenia, that are unexposed to antipsychotics. Some studies support a genetic predisposition to diabetes among people with schizophrenia, suggesting shared genetic risk [66].

### 4.5. Accelerating Aging in Psychotic Disorders

Several researchers argue that the definition of “older adult” in schizophrenia differs from the usual definition. It is well known that patients with schizophrenia suffer premature morbidity and present increased mortality, compared to the general population, and this could be a sign of accelerating physical aging. Indeed, it has been suggested that patients with schizophrenia in their 40s and 50s are medically comparable to the general population in their 60s and 70s [67]. The concept of accelerated biological aging is considered by some as an intrinsic factor in SMI, at least in some individuals. The notion of accelerated biological aging in schizophrenia-spectrum disorders is supported by reports of the detection of certain biomarkers of ageing in those patients, such as leukocyte telomere length and epigenetic ageing [68].

### 4.6. Sedentary Lifestyle and Reduced Physical Activity

Sedentary behavior and low physical activity are highly prevalent in patients with schizophrenia and are independent, yet modifiable, risk factors for cardiovascular disease and premature mortality [69]. In a large-scale survey, Stubbs et al. [70] analyzed data of 204,186 people across 46 low- and middle-income countries, and found that the diagnosis of a psychotic disorder, especially among males, was associated with physical inactivity. Further analysis suggested that low activity rates in patients were mediated by mobility difficulties, self-care difficulties, depressive symptoms, cognitive disturbances, pain and discomfort. In a meta-analysis of 69 studies, it was found that people with SMI engaged in significantly less physical activity than age- and gender-matched healthy controls, and were significantly less likely than matched healthy controls to meet physical activity guidelines. The diagnosis of schizophrenia and antipsychotic medication use were particularly associated with lower physical activity levels in patients [69].

### 4.7. Antipsychotic Medication

Antipsychotic drugs are the cornerstone in treatment of schizophrenia and related psychoses. However, certain effects of these compounds may constitute risk factors for cardiovascular morbidity and mortality, such as the well-documented cardio-metabolic adverse effects [71]. Moreover, the sedative effects of certain antipsychotics [72,73] may further negatively impact the already limited physical activity of patients.

## 5. Other Factors Associated with Elevated Mortality in Schizophrenia-Spectrum Disorders

Patients with schizophrenia have increased risk for physical morbidity, but elevated mortality in those patients may be mediated by factors involving the society and the health system.

### 5.1. Access to Medical Care and Quality of Care

There is evidence that patients with schizophrenia and physical comorbidities may not receive the proper medical care. A recent Danish study found that patients with schizophrenia and incident heart failure were less likely to receive quality care, according to guideline recommendations, compared with other patients with heart failure. Notably, poor psychosocial functioning of patients with schizophrenia was a predictor of less optimal than recommended heart failure care. Those patients experienced a substantially higher risk of 1-year mortality [74]. Moreover, it has been suggested that unrecognized myocardial infarction may be more common in patients with schizophrenia and that the number of patients receiving adequate treatment for previous myocardial infarction is low [75]. Notably, in a previous nationwide register-based study in Sweden it was found that levels of healthcare quality were significantly lower for psychiatric patients [76].

### 5.2. Stigma 

Health outcomes in patients with schizophrenia, may be negatively affected by stigma. Previous research has shown that a large proportion of medical students and health service staff held negative attitudes toward patients with mental illness [77]. More recent research confirmed that stigma toward mental illness is widely spread among health professionals, and may hamper the provision of care and the promotion of mental well-being [78]. Indeed, more than 17% of patients experienced discrimination when treated for physical health problems in a previous cross-sectional survey in 27 countries, and probably this figure undermines the extend of the problem [79]. Perceived discrimination may contribute to the avoidance of medical services by patients with schizophrenia, and is associated with less favorable outcomes when treating the physical symptoms of those patients [79].

**Figure 3:**
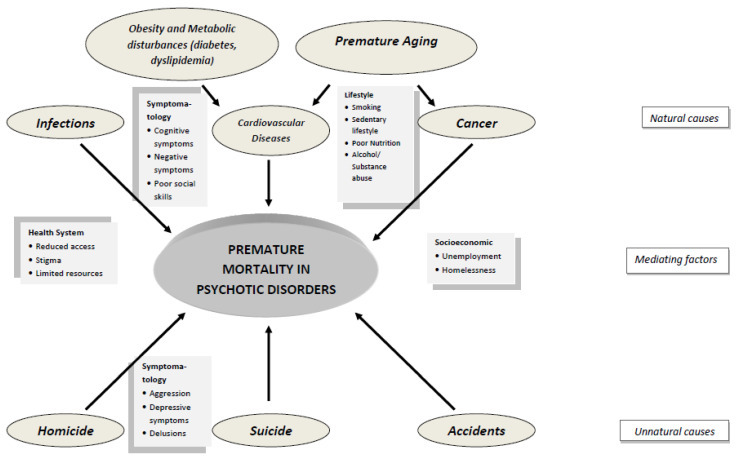
The complex interplay among symptomatology, lifestyle and environment in premature mortality in schizophrenia and related disorders.

All factors that are associated with increased mortality in schizophrenia are summarized in Table 1. The complex interplay of these factors is depicted in Figure 3.

## 6. Protective Factors

Despite the cardiometabolic side effects of antipsychotics, the existing evidence suggests that chronic antipsychotic use is associated with an overall increase in life expectancy of patients [80]. A previous study of death registers in Finland found that antipsychotic use decreased all-cause mortality compared to no antipsychotic use in patients with schizophrenia. Clozapine had the most beneficial profile in this regard [81]. More recently, the 20-year follow-up of the nationwide cohort of 62,250 patients with schizophrenia in Finland confirmed that long-term antipsychotic use does not increase severe physical morbidity in patients with schizophrenia, but rather is associated with substantially decreased mortality, especially among patients treated with clozapine [82]. Most recently, a large 5-year national cohort study in Taiwan that involved 102,964 patients with schizophrenia found that antipsychotic exposure of patients had been associated with better survival outcomes in all-cause mortality, particularly when adequate dosages were used [83]. It is worthy to mention that the use of second-generation long-acting injectable antipsychotics (LAIs) has been associated with the lowest mortality risk in patients with schizophrenia [84], whereas LAIs decreased all-cause mortality and suicide risk in newly diagnosed patients with schizophrenia [85].

Notably, an earlier study in Australia suggested that community treatment orders may reduce mortality among patients with psychiatric disorders. This was partly explained by increased contact with health services in the community [86] and is in line with the aforementioned evidence that antipsychotic treatment is associated with reduced risk of premature mortality in patients with schizophrenia.

## 7. Discussion

Mortality in patients with schizophrenia and related disorders remains high, despite the ongoing advances in our knowledge on these disorders and the progress in treatment. The data that have been presented here highlight the interplay of contributing factors at various levels, that result to physical morbidity and mortality in patients with schizophrenia and related psychoses. Accordingly, interventions to promote physical health and reduce mortality in those patients should be multifaceted.

### 7.1. Interventions for the Reduction of Mortality in Schizophrenia

In a large Canadian study, it was found that although mortality rates among people with schizophrenia have declined over the past 2 decades, there was still a persistent 3-fold relative mortality gap with the general population [35]. Other research has found a widening mortality disparity between those with and without SMI over time [14]. Given the increasing health promotion, public health interventions and improvements in the management of chronic diseases that took place over the last decades, this observation suggests that patients with psychotic disorders may have not fully benefited from the public health strategies that have led to reduced mortality in the general population [35]. This notion is alarming and calls for action to tackling health inequities in patients with psychotic disorders. Table 2 summarizes the interventions that have been studied and the recommendations that have been made accordingly. 

Several of those recommendations place emphasis on physical health monitoring by mental health and primary care professionals, and the patients themselves. However, there is insufficient evidence that physical health monitoring per se is effective in reducing cardiovascular risk in patients with psychotic disorders. Rather, a shift toward primary prevention strategies has been proposed, that includes assertive smoking cessation and dietary programs along with physical exercise interventions. Additionally, it is suggested to avoid long-term prescription of antipsychotics associated with metabolic side effects [88]. However, a very recent meta-analysis found that consistent and long-term use of second-generation antipsychotics (including LAIs, or even clozapine in patients with schizophrenia across all stages of illness can reduce the mortality risk, including that due to cardiovascular disease [89].

In a previous extensive literature review, it was found that the overall strength of the evidence was low for the majority of interventions studied, although the evidence was strongest for interventions to prevent overweight and obesity among persons with SMI. Additionally, the strength of the evidence was strongest for interventions to address tobacco smoking [90]. The contribution of primary healthcare in addressing physical morbidity in SMI should be also highlighted. A recent study in the UK found that comprehensive care plans in primary care for patients with SMI were associated with reduced risk of attending the emergency department, as well as reduced risk of unplanned admission to hospital for physical health problems [91].

### 7.2. Suicide Prevention

**Figure 4:**
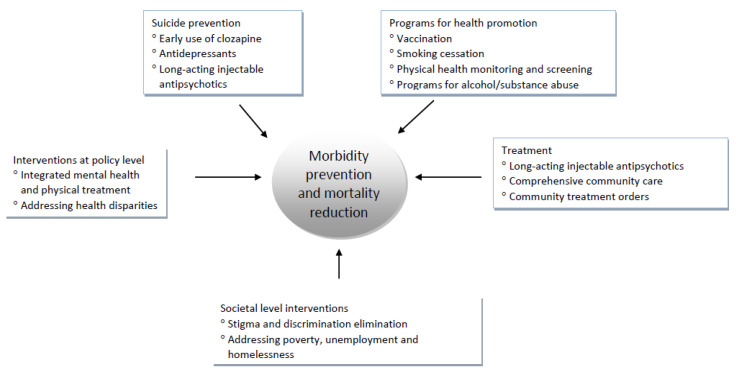
The range of interventions for reducing mortality in schizophrenia.

Suicidal ideation, plans and previous attempts, are as common as 27.7% in outpatients with schizophrenia, and are the strongest predictors of completed suicide [92]. Recognizing suicidal risk factors in schizophrenia is extremely important in suicidal risk assessment, and in this regard a suicide risk formulation has been recently proposed, comprising risk status, risk state, available resources and foreseeable events [93]. Current research suggests that the only reliable protective factor against suicide in patients with schizophrenia is provision of and compliance with comprehensive treatment [26]. Prevention of suicidal behavior in schizophrenia should comprise effective treatment of psychotic symptoms and comorbid depression, as well as management of alcohol/substance misuse [26]. Accordingly, the use of rational psychopharmacological therapy is essential, and may involve the early use of clozapine, which can significantly reduce the risk of suicidality in patients with psychotic disorders, probably through the reduction of depressive symptoms. Concomitant antidepressant medication may be also warranted in this regard [28]. Notably, in a recent nationwide retrospective analysis in the USA it was found a small but negative association between the number of community mental health centers and deaths by suicide. The authors suggested that declines in the number of those services from 2014 to 2017 might have been associated with the 6% of the national increase in suicide. Conceivably, community mental health services may be an important component of suicide prevention efforts [94], probably because they enable patients’ engagement to treatment. The broad range of interventions for the reduction of mortality in schizophrenia-spectrum disorders is summarized in Figure 4.

### 7.3. Implications for Healthcare Policy and Future Research

Future research should address the factors that are associated with increased mortality in patients with psychotic disorders and the most effective interventions, according to the social, cultural, geographic and economic background of the patients. There seem to be differences in the causes of mortality between developed and developing countries. For instance, infections may be more relevant in some developing countries than cardiovascular events [25,95], and interventions need to be adjusted accordingly. It is thus encouraging that developing countries fund research on the development of community level interventions for the management of physical morbidity in patients with SMI [96]. Future research should also address mortality in patients with schizophrenia spectrum disorders in the rural context. Although research on rural patients with schizophrenia spectrum disorders is limited [97], it has been shown that there may be significant inequalities in patient care between rural and urban settings [98]. It is thus important to have reliable data on patients’ mortality that would guide the planning and implementation of preventive strategies and programs, in collaboration with primary healthcare professionals. 

Interventions should target all patient-related modifiable factors, but also factors related to health services. That is, interventions toward patients’ unhealthy lifestyle, including smoking, substance use, physical inactivity and poor diet, as well as toward treatment side effects, should be accompanied by relative changes in health policy. A previous Swedish national cohort study of 6,097,834 adults, including 8277 patients with schizophrenia, with a 7-year follow-up, found that among people who died from ischemic heart disease or cancer, those with schizophrenia were significantly less likely than others to have been previously diagnosed with these conditions. Notably, among people who were previously diagnosed, those with schizophrenia had only a modestly greater mortality risk from ischemic heart disease and no increased cancer mortality risk compared with the rest of the population [99]. Similarly, a recent meta-analysis of 47 observational studies found that patients with schizophrenia receive less screening and lower-quality treatment for cardiovascular disorders. The authors stressed the importance to address underprescribing of appropriate medications and underutilization of diagnostic and therapeutic procedures in this patient population [100]. Conceivably, it is crucial to improve the access to physical healthcare for patients with schizophrenia, if we are to properly diagnose physical morbidity and address excess mortality. Yet, fragmentation in coordinated care between physical and mental health settings, as well as between primary care physicians and tertiary hospitals has been highlighted in the literature, and may mediate poorer physical health outcomes in patients with schizophrenia-spectrum disorders [101]. In this regard, integrated care has emerged as a way to address the physical health needs of those patients. This model of care may involve co-location of physical and mental health services, liaison services, shared protocols and information sharing systems, among others [101,102]. 

In a recent population-wide data linkage in New South Wales, Australia, the authors compared the potentially preventable hospitalizations for physical illness of 178,009 patients with SMI that attended community mental health services with the respective general population rates. It was found that patients had higher rates of potentially preventable admissions, whereas the length of hospital stay was also significantly higher in those patients. Importantly, vaccine-preventable infections and chronic medical conditions, such as chronic obstructive pulmonary disease and diabetes complications, accounted for a large proportion of those hospitalizations [103]. These findings highlight the importance of adequate monitoring of chronic medical conditions in patients with SMI, as well as vaccination, particularly in the era of the COVID-19 pandemic. 

It is important all interventions to address mortality in schizophrenia and related syndromes to take place as early as possible in the course of the patients’ treatment. A recent meta-analysis found higher relative mortality in younger age groups, whereas the incidence of suicide was also found to be elevated in younger patients and during the first years after diagnosis [21]. It has been argued that clinicians should be aware of the high risk of cardiovascular morbidity and mortality in patients with a first episode of psychosis receiving antipsychotics, and that monitoring weight and metabolic changes over time should be mandatory [104]. Those patients may be treated in specialized early intervention services [105], conceivably these services should be properly designed and collaborate with other physicians to address physical health problems alongside psychiatric symptoms. 

## 8. Conclusions

Schizophrenia and related syndromes are still life-shortening and the longevity gap is only increasing, compared to the general population, suggesting that patients have not fully benefited by progress in medicine and treatment. Suicide and other unnatural causes of death are still challenging for clinicians and the health system, but most deaths are due to preventable physical morbidity, such as cardiovascular disease, infections and cancer, thus providing opportunities for multi-level interventions.

Mortality from natural causes is mediated by several factors involving the patient, the society and the health system that act synergistically, thus interventions should be multifaceted. Nation-wide programs for patients with schizophrenia should involve smoking cessation, promote vaccination and address alcohol/substance abuse. Initiations to eliminate health disparities in those patients and improve access to screening and quality healthcare should be undertaken, and integrated care for mental and physical health has emerged as a way to address disparities in care.

Long-term antipsychotic treatment may have a protective effect against mortality thus mental health services should prioritize patients’ adherence to treatment, and monitor cardiometabolic side effects of antipsychotics to optimize treatment regimen. Early use of clozapine and LAIs may also reduce mortality from cardiovascular disease and suicide, whereas antidepressants are also helpful in this regard. Comprehensive, interdisciplinary community mental healthcare should be enabled for all patients, and may be more relevant for those residing in rural areas. Further research on interventions that would address premature mortality in schizophrenia-spectrum disorders is urgently needed to improve knowledge and to guide clinical practice and mental health policy.

## Figures and Tables

**Table 1:** Factors associated with increased mortality by natural causes in patients with schizophrenia-spectrum disorders [22].

Patient-related factors	Unhealthy life style Smoking Alcohol/substance use Sedentary life style Poor nutritional habits Poor compliance to medical instructions Poor treatment adherence Low health literacy Sexual and other risky behaviors Stigma
Symptomatology	Cognitive dysfunction Negative symptoms Poor communicative and social skills
Treatment-related factors	Medication adverse effects Weight gain Hyperglycemia/diabetes mellitus Dyslipidemia Metabolic syndrome Cardiovascular events Sedation
Health services-related factors	Inadequate health care Negative attitudes of primary care personnel Limited knowledge of psychiatrists on general health issues Stigma Inadequate liaison of services Lack of resources
Other disease-related factors	Inter-relationship between schizophrenia and diabetes mellitus Premature aging Reduced pain perception
Socioeconomic factors	Unemployment Homelessness

**Table 2:** Interventions for reducing mortality caused by preventable physical morbidity in patients with schizophrenia-spectrum disorders [22,87].

At patient level	Patients’ encouragement to monitor simple measures, such as weight, diet, and exercise programs Appropriate monitoring of antipsychotic treatment and switching in cases of excessive weight gain and metabolic disturbances
At service level	Efforts to increase awareness of the problem among primary care and mental health providers Addressing the inadequate treatment of physical health problems in mentally ill patients by primary care physicians Education and training of psychiatrists in physical health issues Adherence of psychiatrists to the guidelines regarding physical health monitoring in mentally ill patients
At policy level	Appropriate designation and equipment of community mental health services and outpatient clinics to enable proper assessment of physical health monitoring Legislative changes to minimize discrimination against psychiatric patients Reduction of health disparities Programs to eliminate unemployment and poverty Further research on physical health of psychiatric patients

## Data Availability

Not applicable.
